# Reward-Predictive Neural Activities in Striatal Striosome Compartments

**DOI:** 10.1523/ENEURO.0367-17.2018

**Published:** 2018-02-05

**Authors:** Tomohiko Yoshizawa, Makoto Ito, Kenji Doya

**Affiliations:** 1 Neural Computation Unit, Okinawa Institute of Science and Technology Graduate University, Onna-son, Kunigami-gun, Okinawa 904-0412, Japan; 2 Development Department, Progress Technologies Inc, Koto-ku, Tokyo 135-0064, Japan

**Keywords:** calcium imaging, reinforcement learning, reward, striatum, striosome, value

## Abstract

The striatum has been shown to play a critical role in reward prediction. It is composed of two neurochemically and anatomically distinct compartments known as the striosomes and the matrix. The striosomes comprise only about 15% of the striatum by volume and are distributed mosaically therein. Accordingly, it has been difficult to identify striosomal neurons in electrophysiological recordings and it has been unclear whether striosomal neurons, which project to midbrain dopaminergic neurons, engage in reward prediction. In this study, we utilized a mouse line (Sepw1-NP67) selectively expressing Cre in striosomal neurons, combined with endoscopic *in vivo* calcium imaging to selectively record activities of striosomal neurons during an odor-conditioning task. As mice learned the task, striosomal neurons in the dorsomedial striatum (DMS) showed predictive activities to odor cues that were associated with water rewards or aversive air puffs. These activities were proportional to the expected reward or air-puff intensity. Intriguingly, repeated recordings of the same striosomal neurons over a period of weeks revealed that predictive activities were learning-stage specific. That is, these activities disappeared after continuous training. Furthermore, presentations of rewards or air puffs activated some striosomal neurons. These findings suggest that the striosomes participate in reward prediction with learning stage-specific neural ensembles, and that they also send reward and aversive signals to dopaminergic neurons.

## Visual Abstract

**Figure:**
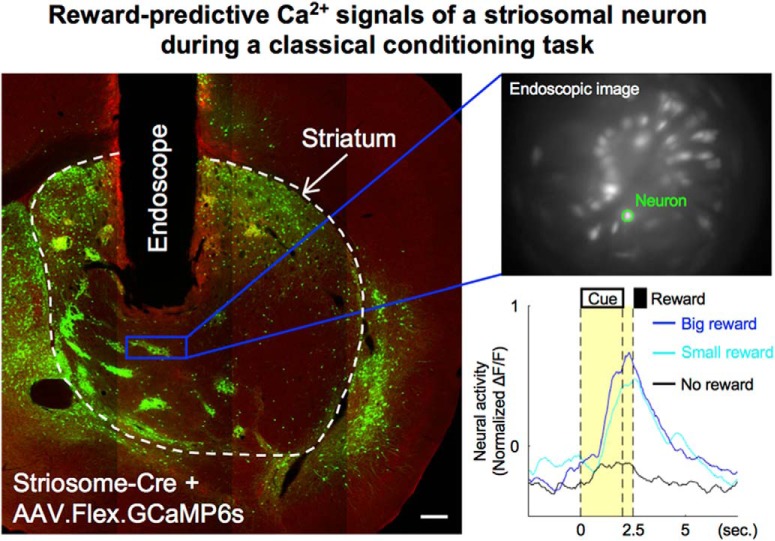

## Significance Statement

Striosomes are striatal compartments that directly project to midbrain dopaminergic neurons. By using an endoscopic *in vivo* calcium imaging device and a striosome-Cre mouse line, we succeeded in selective recoding of striosomal neurons during a classical conditioning task and discovered reward-predictive activities proportional to the expected reward amount. Interestingly, most reward-predictive activities of striosomal neurons were observed only in early or late stage of learning. In addition, some striosomal neurons were directly activated by reward experiences. These results suggest that striosomal neurons transmit both expected and acquired reward signals to dopaminergic neurons.

## Introduction

The striatum consists of two neurochemically and anatomically distinct compartments: the striosomes (also known as patches), which are rich in μ-opioid receptors (MORs), receive inputs from the limbic cortex, and project monosynaptically to midbrain dopaminergic neurons, and the matrix, which receives inputs from the sensorimotor and associative cortices (Jimenez-Castellanos and Graybiel, 1987; Gerfen, 1989; Eblen and Graybiel, 1995; Kincaid and Wilson, 1996). Many lines of research, including functional brain imaging (O'Doherty et al., 2004; Tanaka et al., 2004) and neural recording (Samejima et al., 2005; Ito and Doya, 2009, 2015; Kim et al., 2009) have demonstrate that the striatum plays a critical role in decision-making and reinforcement learning. In the process of reinforcement learning, prediction of forthcoming rewards from the present sensory state and possible actions such as “state value” and “action value,” respectively, comprise the basis for learning and action selection (Sutton and Barto, 1998). These values are updated by a reward-prediction error, defined as the discrepancy between the predicted and actual rewards. The striatum is a major cortical-input site of the basal ganglia and also receives inputs from midbrain dopaminergic neurons encoding the reward-prediction error (Schultz et al., 1997). Cortico-striatal synapses show dopamine-dependent plasticity that is suitable for reinforcement learning (Reynolds et al., 2001). From these observations, the striatum has been hypothesized as the brain region that predicts future rewards as state or action values (Kawagoe et al., 1998; Shidara et al., 1998; Pagnoni et al., 2002; O'Doherty et al., 2004). In fact, electrophysiological studies have shown that striatal neurons encode state or action values (Samejima et al., 2005; Pasquereau et al., 2007; Lau and Glimcher, 2008; Kim et al., 2009; Ito and Doya, 2009, 2015), but they could not identify whether recorded neurons belonged to striosomes or matrix, because these compartments form a mosaic-like structure (Pert et al., 1976; Graybiel and Ragsdale, 1978; Herkenham and Pert, 1981). Because striosomal neurons comprise only ∼15% of striatal neurons, it is particularly unclear whether striosomal neurons engage in reward prediction. It is important to characterize their activities during reward-based learning because almost all striatal neurons directly projecting to midbrain dopaminergic neurons belong to striosome compartments (Jiménez-Castellanos and Graybiel, 1989; Tokuno et al., 2002; Fujiyama et al., 2011; Watabe-Uchida et al., 2012).

Recently, a transgenic mouse line became available that selectively expresses Cre protein, which is a site-specific DNA recombinase, in striosomal neurons (Gerfen et al., 2013; Smith et al., 2016). In combination with optical neural imaging, it is possible to image deep brain structures using endoscopic microscopes (Ghosh et al., 2011; Ziv et al., 2013; Resendez et al., 2016). In this study, to test whether striosomal neurons show reward-predictive activities, we recorded activities of neurons in striosomes during classical conditioning using endoscopic *in vivo* calcium imaging of transgenic mice with selective calcium indicator expression in their striosomal neurons.

## Materials and Methods

### Subjects

Male Sepw1-NP67 (Gerfen et al., 2013) mice (*n* = 8; 25–35 g body weight; 8–12 weeks old) were housed individually under a 12/12 h light/dark cycle (lights on at 7 A.M.; off at 7 P.M.). Experiments were performed during the light phase. Water was restricted to 1–2 ml/d for two weeks before experimental initiation and during the experimental period. Food was provided *ad libitum* for the entire period. The Okinawa Institute of Science and Technology Graduate University Animal Research Committee approved the study.

### Surgery

Mice were anesthetized with isoflurane (1.0–3.0%) and placed in a stereotaxic frame. The skull was exposed, a hole (diameter: 1.0 mm) was drilled in the skull, and the dura was removed over the imaging site. For calcium imaging, 0.4–0.6 µl of AAV2/9.Syn.Flex.GCaMP6s (*n* = 5 mice) or AAV2/9.Syn.GCaMP6s (*n* = 3, Penn Vector Core) were injected into the striatum (AP, +0.50 mm; ML, ±1.75 mm; DV, 2.85 mm from brain surface). Three weeks after virus injection, an endoscope (GRIN lens; PartID, 130-000151; diameter, 0.5 mm; length, 6.1 mm; Inscopix) with a custom endoscope holder was slowly implanted at the following coordinates: AP, +0.50 mm; ML, ±1.75 mm; DV, 2.60 mm. The endoscope was fixed with UV adhesive (LOCTITE 4305, Henkel) and clear dental cement (Super bond, Sun Medical) and protected by a PCR tube. A head plate (CF-10, NARISHIGE) was fixed with pink dental cement. Two to four weeks after endoscope implantation, awake mice were head-fixed with a head plate holder. A baseplate (Part ID: 100-000279; Inscopix) attached to the miniature microscope was positioned above the endoscope. The focal plane (100–300 μm working distance) was adjusted until neuronal structures and GCaMP6s responses were clearly observed. After mice were anesthetized with isoflurane, the baseplate was fixed with black-painted dental cement (CLEARFIL MAJESTY ES Flow; Kuraray Noritake Dental) and a baseplate cover (part ID: 100-000241; Inscopix) was secured to the baseplate with a set screw to protect the lens until imaging.

### Behavioral task

Mice were head-fixed using the head plate and habituated for 3–5 d before task training. A custom-built olfactometer (O’Hara) delivered a 1:9 mixture of air saturated with one of four odors (isoamyl acetate, citral, eugenol, or (-)carvone) and clean air. The olfactometer constantly delivered clean air during inter-trial intervals (ITIs). ITIs were randomly selected from 10 to 20 s. In each trial, we delivered one of four odors, selected pseudorandomly, for 2 s, followed by a delay of 0.5 s and an outcome. Each odor was associated with a different outcome: a big drop of water (4 µl), a small drop of water (2 µl), no outcome, or an air puff delivered to the animal’s face. These outcomes were randomly omitted with a 20% probability. The combination of odor and outcome differed for different mice. A daily session consisted of 100 trials. Licks were detected by interruptions of an infrared beam placed in front of the water tube. 1 g of water gel (HydroGel; ClearH_2_O) was provided after daily sessions.

### Calcium imaging

In each daily session, we first head-fixed mice using the head plate and holder. Then we connected the microscope to the magnetic baseplate, and fixed it in place with the baseplate set screw. Fluorescence images were acquired at 20 fps with LED power at 20% of 1.2 mW/mm^2^ maximum and the image sensor gain at 1.0–4.0 before A/D conversion. To compare calcium activity in different sessions, image acquisition parameters were held constant for each mouse across days. An external signal (5V TTL) from the control device triggered the start or end of recording. Neural activities in each trial were recorded from 2.5 s before odor onset to 5 s after unconditioned stimulus (US) onset (total: 10 s/trial) to minimize photo toxicity.

### Image processing

All image processing was performed in Mosaic (version 1.1.3; Inscopix) and Matlab (version 2016b; Mathworks). First, the raw image of each frame was translated into a 16-bit tiff image. To reduce data size and processing time, spatial down-sampling (spatial binning factor: 4) was applied to each tiff image. After image sequences of all trials for each session were concatenated, a motion correction process was applied to remove movement artefacts and to compensate for shifts in microscope positioning. After removing the post-registration black borders, average fluorescence F was calculated over the whole motion-corrected image sequence and percentage-change-over-baseline (ΔF/F = (F*_n_* – F)/F) images were generated for each frame. Here, F*_n_* was the motion-corrected image at *n*-th frame. Finally, ΔF/F image sequences of all sessions for each animal were concatenated, and temporally down-sampled (temporal binning factor: 4), then spatial filters to extract activities of single neurons were calculated with a cell-sorting algorithm using independent and principal component analyses (Mukamel et al., 2009).

### Extraction of calcium signals and event detection

To extract calcium signals of each neuron at 20 Hz, spatial filters were applied to the original ΔF/F image sequence of each session. The extracted calcium signal of each neuron was normalized to: mean = 0, variance = 1 (normalized ΔF/F) for each session because the expression levels of GCaMP6s could have differed between neurons and sessions. Then, “Ca^2+^ events” (Okuyama et al., 2016; Kirschen et al., 2017) were detected by applying the following procedure. For the normalized ΔF/F trace in each trial *i*, all local maxima were detected and for *j*-th local maximum (*M_ij_*), the preceding local minimum (*m_ij_*) was registered. When the difference (Δ*m_ij_* = *M_ij_*– *m_ij_*) between the local maximum and the preceding minimum exceeded a threshold (4× the median absolute deviation, 4 MAD), Δ*m_ij_* was registered as a Ca^2+^ event of amplitude (*y_ik_*) at the midpoint time (*t_ik_*) between the time of *M_ij_* and *m_ij_*, where *k* is the index of the event in a trial.

### Experimental design and statistical analysis

To show that a neuron encodes outcomes expected from odor stimuli rather than odor natures, changing of CS-US combinations between mice is effective. Therefore, we needed at least two mice each from the striosome and control groups. We actually used five and three mice from the striosome and control groups, respectively, to collect enough samples to analyze their properties.

Two-sample *t* tests were employed for statistical tests for frequencies of licking or Ca^2+^ events between task conditions. To evaluate neural representations of behavioral variables, we conducted regression analyses of Ca^2+^ events during the CS-delay period (2.5 s between CS onset and US onset) and the US period (2.5 s following US onset). Regression analysis employed the variables licking frequency (*Lick*), prediction of reward (*Vr*), air puff (*Va*), delivery of reward (*Rwd*), and air puff (*Air*). The variables *Vr* and *Rwd* took one of three levels: 0 (0 µl), 0.5 (2 µl), and 1 (4 µl) while *Va* and *Air* took 0 or 1. Note that *Rwd* and *Air* took 0 in omission trials, so that they were different from *Vr* and *Va*. The sum of the amplitudes of all Ca^2+^ events during the CS-delay or US period of *i*-th trial was registered as, *y*(*i*,CS) and *y*(*i*,US). First, to remove the effects of licking on neural activities, we performed the following regression analysis and obtained the residual activities *z*:

$$y(i,s)=\beta_{0}+\beta_{\mathrm{Lick}}\mathrm{Lick}(i,s)+z(i,s)$$

where *s* = CS or US denotes the time period. We then analyzed residual activities in the CS and US periods using the following regression models.

For big, small, and no reward conditions:

$$z(i,\mathrm{CS})=\beta_{1}+\beta_{\mathrm{Vr}}\mathrm{Vr}(i)$$

$$z(i,\mathrm{US})=\beta_{2}+\beta_{\mathrm{Rwd}}\mathrm{Rwd}(i)$$

For air-puff and no reward conditions:

$$z(i,\mathrm{CS})=\beta_{3}+\beta_{\mathrm{Va}}\mathrm{Va}(i)$$

$$z(i,\mathrm{US})=\beta_{4}+\beta_{\mathrm{Air}}\mathrm{Air}(i)$$

When the *p* value of the regression coefficient was <0.05, we concluded that neural activity and the explanatory variable were significantly correlated, χ^2^ tests were used for comparison of proportions of predictive/responsive neurons between groups or stages.

For the decoding analysis, we used *n* = 1–10 simultaneously recorded neurons. Since the number of simultaneously recorded neurons differed between mice, we randomly selected *n* neurons from simultaneously recorded populations and regressed *Vr* or *Va*, and *Rwd* or *Air* with the sum of amplitudes of Ca^2+^ events of them during the CS-delay and US period.

For big, small, and no reward conditions:

$$\mathrm{Vr}(i)=w_{\mathrm{Vr},0}+\overset{n}{\underset{j=1}{\sum }}w_{\mathrm{Vr},j}x_{j}(i,\mathrm{CS})$$

$$\mathrm{Rwd}(i)=w_{\mathrm{Rwd},0}+\overset{n}{\underset{j=1}{\sum }}w_{\mathrm{Rwd},j}x_{j}(i,\mathrm{US})$$

For air-puff and no reward conditions:

$$\mathrm{Va}(i)=w_{\mathrm{Va},0}+\overset{n}{\underset{j=1}{\sum }}w_{\mathrm{Va},j}x_{j}(i,\mathrm{CS})$$

$$\mathrm{Air}(i)=w_{\mathrm{Air},0}+\overset{n}{\underset{j=1}{\sum }}w_{\mathrm{Air},j}x_{j}(i,\mathrm{US})$$

where *x_j_*(*i*,CS) and *x_j_*(*i*,US) are the sum of amplitudes Ca^2+^ events during the CS-delay and US period, and ${w_{Vr,j},w_{Rwd,j},w_{Va,j},w}_{Air,j}$are weights for *j*-th neuron out of *n* neurons. After 100 iterations of these procedures for each population size *n*, we averaged MSEs of each group's mouse to compare the population coding of expected and actual US between two groups, and tested them by paired *t* test.

### Immunohistochemistry

We adapted an immunohistochemical protocol for identifying striosomes in rats (Jedynak et al., 2012) for use with mice. After all experiments were completed, mice were deeply anesthetized with pentobarbital sodium and then perfused with 4% paraformaldehyde (PFA). Brains were carefully removed so that endoscopes would not cause tissue damage, post-fixed in 4% PFA at 4°C overnight, and then transferred to a 30% sucrose/PBS solution at 4°C until brains sank to the bottom. Coronal or horizontal sections were cut at 30 µm on an electrofreeze microtome (REM-710; Yamato) and stored in wells containing PBS at 4°C. Free-floating sections were washed in PBS for 5 min and placed in blocking buffer (5% normal donkey serum and 0.4% Triton X-100 in PBS) for 2 h at room temperature (RT). Sections were simultaneously incubated in primary antibody-rabbit anti-MOR (ab10275; Abcam) diluted 1:500 in blocking buffer, for 48 h at 4°C. Two days later, sections were washed 6x for 10 min in PBS and placed in blocking buffer for 1 h at RT. Sections were simultaneously incubated in secondary antibody donkey anti-rabbit (Alexa Fluor 594; Invitrogen) diluted 1:250 in blocking buffer for 2 h at RT. Sections were washed 6x for 10 min in PBS, mounted on glass slides and coverslipped with VECTASHIELD Mounting Medium with DAPI (Vector Laboratories). To inspect stained tissue, a confocal microscope (LSM780; Carl Zeiss) was used and pictures were taken using ZEN software.

## Results

### Spout-licking behavior during odor conditioning

**Figure 1.**
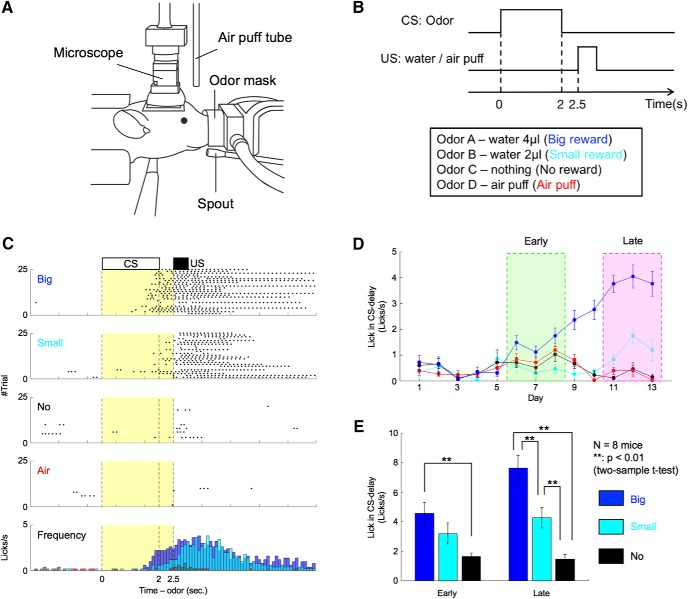
Mice showed odor-induced reward-predictive licking behavior proportional to expected reward size. ***A***, Schematic illustration of the behavioral apparatus. Mice were restricted, head and body, by the metal frame and tube. The odor mask, water spout, and air-puff tube were set in front of their noses, mouths, and eyes. Spout-licking behaviors were monitored using an infrared sensor. The miniature microscope was mounted on their heads. ***B***, Time sequence of a classical conditioning task. ***C***, An example of reward-predictive spout-licking behaviors after sufficient learning. In trials of reward conditions, spout-licking behaviors started during odor presentation periods. Black dots indicate spout-licking behaviors. Yellow areas show CS-delay periods. ***D***, Daily changes of spout-licking frequency during CS-delay periods of the mouse illustrated in ***C***. Early and late stages were defined based on the appearance of reward-predictive licking. Error bars indicate SEs. ***E***, Average spout-licking frequencies during CS-delay periods of all eight mice. Error bars indicate SEs.

We employed classical odor conditioning, a standard reward-based learning task for rodents (Oyama et al., 2010; Cohen et al., 2012). Water-deprived mice were classically conditioned with different odor cues predicting water (reward) or air puffs (aversive stimuli) under head-restrained conditions (Fig. 1*A*). Daily training sessions were composed of 100 trials. Each trial began with a conditioned stimulus (CS; odor, 2 s), followed by a delay period (0.5 s) and an US (water 4 µl/water 2 µl/air puff/nothing; Fig. 1*B*). For each mouse, the CS was randomly selected from four odor cues that the mouse had to associate with different US, and the CS was fixed for all days. The combination of CS-US was varied among mice. To evaluate reward-prediction performances of the mice, we counted the number of licks toward the water-delivery spout.

In early training, mice licked the spout immediately after reward onset in some trials. After days of conditioning, they began licking during the CS-delay period before rewards arrived (Fig. 1*C*). To detect stages of learning, we quantified each mouse’s mean daily licking frequency during the CS-delay period. Licking frequency showed no significant differences between the four odor conditions until day 5. Then commencing at day 6, it became significantly higher in the big-reward condition than in other conditions (Fig. 1*D*). By day 11, licking frequencies in big-reward, small-reward, and no-reward conditions differed significantly. Although the numbers of days for CS-US learning differed depending on the mouse, all eight mice displayed similar behavior. Therefore, we defined two learning stages: “early stage,” comprising the first 3 d that licking frequency in the CS-delay period became significantly faster in the big-reward condition than in the no-reward condition (*p* < 0.05, two-sample *t* test), and “late stage,” comprising the first 3 d that licking frequencies during the CS-delay period in big-reward, small-reward, and no-reward conditions all differed significantly (*p* < 0.05). The number of days from training initiation to the early stage was 4.6 ± 0.71 (average ± SE) and to the late stage was 12 ± 1.1. Licking frequency during the CS-delay period increased monotonically with reward size in both stages (Fig. 1*E*). This result indicates that mice predicted forthcoming rewards from odor stimuli by learning CS-US associations.

### Selective *in vivo* calcium imaging of neurons in striosomes

**Figure 2.**
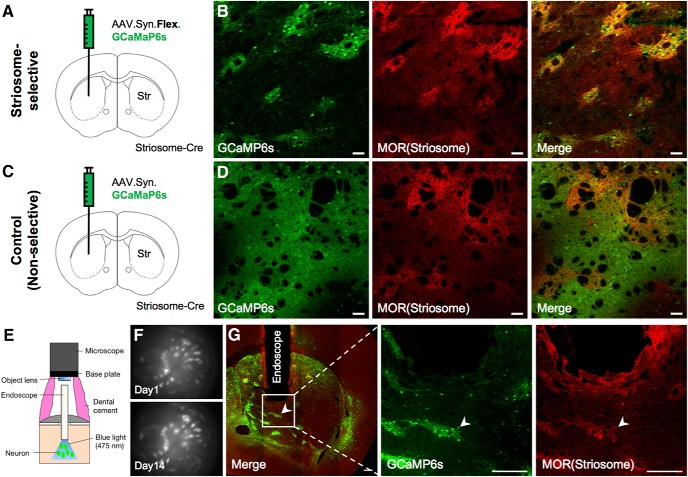
An endoscopic microscope was used for selective *in vivo* calcium imaging of striosomal neurons in the striata of Sepw1-NP67 mice expressing Cre-dependent GCaMP6s. ***A***, Striosome group. To express GCaMP6s selectively in striosomal neurons, AAV.Syn.Flex.GCaMP6s was injected into the DMS. ***B***, GCaMP6s (green) was selectively expressed in striosomes (red) three weeks after virus injection. Scale bar: 50 µm. ***C***, Control group. To express GCaMP6s in both striosomes and matrix, AAV.Syn.GCaMP6s was injected to DMS. ***D***, GCaMP6s expressed in both striosomes and matrix three weeks after virus injection. ***E***, Schematic illustration of endoscopic *in vivo* calcium imaging. ***F***, Averaged fluorescence images recorded by miniature microscope. White dots indicate neurons. The same neurons in striosomes were stably observed over two weeks. ***G***, Images showing endoscope placement and Cre-dependent GCaMP6s-expressing neurons within the striatum. The focal plane in tissue is 250–300 µm from the bottom of the endoscope, as indicated by the white arrow heads. Scale bar: 200 µm.

We used transgenic mice (Sepw1-NP67) expressing Cre selectively in their striosomal neurons (Gerfen et al., 2013; Crittenden et al., 2016; Smith et al., 2016). To express GCaMP6s selectively in striosomal neurons using the Cre-loxP system, AAV2/9.Syn.Flex.GCaMP6s was injected unilaterally (left hemisphere: two mice, right hemisphere: three mice) into the dorsomedial striatum (DMS) of transgenic mice (striosome group; Fig. 2*A*). MOR immunohistochemistry of virus-injected brain slices confirmed that GCaMP6s was selectively expressed in striosomes (Fig. 2*B*). We also prepared mice expressing GCaMP6s in both striosomes and matrix as the control group by injecting AAV2/9.Syn.GCaMP6s (not containing the loxP sequences, left hemisphere: two mice, right hemisphere: one mouse) to the DMS (Fig. 2*C*,*D*).

An endoscope (GRIN lens, diameter: 0.5 mm) was implanted into the DMS, and neural activities were recorded through the endoscope using a miniature microscope integrating an LED light source and an image sensor (Ghosh et al., 2011; Fig. 2*E*). 122 neurons were recorded from five mice in the striosome group and 83 neurons from three mice in the control group. On average, we were able to simultaneously record 24 neurons (maximum 45) from one mouse in the striosome group and 28 neurons on average (maximum 36) in the control group. Because the advantage of this imaging method is that we can continuously observe the same neurons for several weeks (Ziv et al., 2013; Resendez et al., 2016), calcium imaging was performed in all mice every day from the first to the final day of behavioral experiments (Fig. 2*F*). We measured fluorescence intensity of each neuron during a resting state (for 2.5 s before odor onset in each trial) to check changes GCaMP6s expression level. Although 7% and 8% maximum increases in the median rate of change of fluorescence intensity were observed in the striosome and control groups, respectively, differences between sessions had no significant effect on the rate of change in either group (striosome: *p* = 0.69, control: *p* = 0.64, Kruskal–Wallis test). This indicates that neural activities were stably recorded throughout early and late stages.

After the imaging experiment, we made coronal brain slices including the trace of the endoscope and checked GCaMP6s expression and MOR immunohistochemistry. In all five mice of the striosome group, we confirmed that the GCaMP6s-expressing neurons were located within the working distance of the endoscope (250–300 μm) and that they were included in the MOR-positive striosome compartments (Fig. 2*G*).

### Reward-predictive neural activities

**Figure 3.**
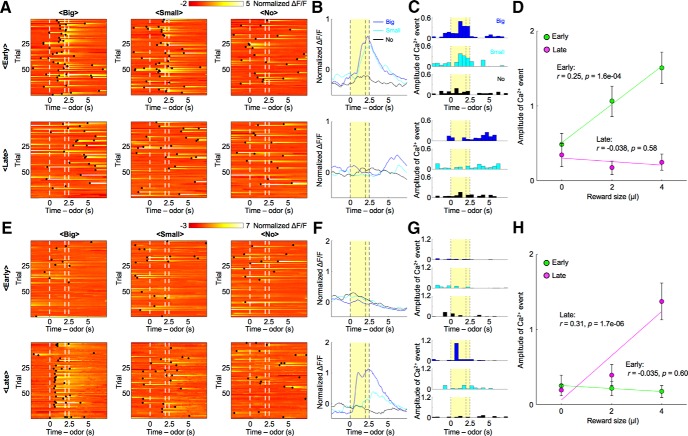
Reward-associated odors activated striosomal neurons in a specific learning stage. ***A***, Normalized ΔF/F of a striosomal neuron showing reward-predictive activity specifically in the early stage. Black dots indicate detected Ca^2+^ events. ***B***, ***C***, Averaged ΔF/F and Ca^2+^ events of the striosomal neuron illustrated in ***A***. Yellow areas show the CS-delay period. ***D***, Amplitudes of CS-delay period Ca^2+^ events of the striosomal neuron illustrated in ***A*** were averaged over trials and plotted against reward size. In the early stage, Ca^2+^ events show a positive correlation with reward size (*r* = 0.25, *p* = 1.6e-04). On the other hand, this correlation disappeared in the late stage (*r* = -0.038, *p* = 0.58). Error bars and lines indicate SEs and regression lines. ***E***, Normalized ΔF/F of another striosomal neuron showing reward-predictive activity specifically in the late stage. ***F***, ***G***, Averaged ΔF/F and Ca^2+^ events of the striosomal neuron illustrated in ***E***. ***H***, Amplitudes of CS-delay period Ca^2+^ events of the striosomal neuron illustrated in ***E*** were averaged over trials and plotted against the reward size. In the early stage, Ca^2+^ events show no significant correlation with reward size (*r* = −0.035, *p* = 0.60). However, a positive correlation was observed in the late stage (*r* = 0.31, *p* = 1.7e-06).

We first examined responses of striosomal neurons to odor stimuli. After normalizing the ΔF/F trace of recoded neurons (normalized ΔF/F), we detected Ca^2+^ events (Okuyama et al., 2016; Kirschen et al., 2017), which estimate the strength of neural activity while taking into account the slow decay time of GCaMP6s (Chen et al., 2013; see Materials and Methods). In the early stage, the normalized ΔF/F of a representative striosomal neuron (Fig. 3*A*,*B*) rose with the presentation of odor stimuli associated with the big reward, whereas no rise was observed in the no-reward condition. The sum of amplitudes of Ca^2+^ events during the CS-delay period in the early stage was significantly larger in the big-reward condition than in the no-reward condition (*p* = 1.2e-04, two-sample *t* test, Fig. 3*C*), while the amplitude in the late stage displayed no significant difference between the big-reward condition and the no-reward condition (*p* = 0.61), as the response to the odor stimulus associated with the big reward became weak. The amplitude correlated positively with forthcoming reward size in the early stage (*r* = 0.25, *p* = 1.6e-04; Fig. 3*D*), but not in the late stage (*r* = −0.038, *p* = 0.58).

In contrast, the sum of amplitudes of Ca^2+^ events in another striosomal neuron during the CS-delay period in the early stage showed no significant difference between the big-reward condition and the no-reward condition (*p* = 0.62; Fig. 3*E–G*), while the response in the late stage was significantly larger in the big-reward condition (*p* = 8.2e-06). The amplitude did not significantly correlate with forthcoming reward size in the early stage (*r* = −0.035, *p* = 0.60; Fig. 3*H*), but positively in the late stage (*r* = 0.31, *p* = 1.7e-06). Neurons in which the sum of amplitudes of Ca^2+^ events during the CS-delay period correlated with forthcoming reward size in one of the learning stages were found in the control group as well.

**Figure 4.**
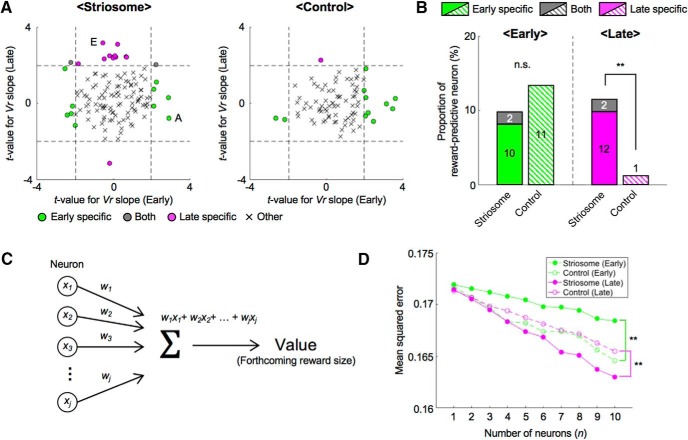
During each learning stage, different neural ensembles participated in reward prediction and population coding of expected reward differed between two groups. ***A***, To remove effects of motor behavior on neural activities, we first performed a regression analysis of the sum of amplitudes of Ca^2+^ events during the CS-delay period with frequencies of licking. Then we analyzed the residual component using prediction of reward (*Vr*). Scatter plots of t-values for regression coefficients of *Vr* in each learning stage. Dashed lines indicate levels of significant *Vr* slope at *p* = 0.05. Letters A and E indicate the example neurons in Figure 3*A*,*E*. ***B***, Proportions of reward-predictive neurons in each learning stage. Numbers in bars indicate actual counts of reward-predictive neurons; **p < 0.01, n.s.: *p* ≥ 0.05, χ^2^ test. ***C***, Schematic illustration of neural decoding analysis. Forthcoming reward size was estimated from the sum of weighted neuronal activities. *x_j_*: sum of amplitudes of Ca^2+^ events during the CS-delay period. *w_j_*: weight for *j*-th neuron out of *n* neurons. ***D***, MSEs between actual and decoded reward sizes at each number of neurons used for analyses; **p < 0.01, paired *t* test.

To quantify proportions of reward-predictive neurons in the striosome, we performed a regression analysis of the sum of amplitudes of Ca^2+^ events during the CS-delay period. To eliminate neural activities directly related to licking movements, we first conducted a regression analysis with licking frequencies and then analyzed residual components with the reward (*Vr*) predicted from the odor cues (see Materials and Methods). In most neurons of both striosome and control groups, reward-predictive activities that had significant regression coefficients to *Vr* were observed specifically in the early or the late stage (Fig. 4*A*); 8% of striosomal neurons (10 of 122) and 13% of control neurons (11 of 83) were reward-predictive in the early stage, but not in the late stage. On the other hand, 10% of striosomal neurons (12 of 122) and 1% of control neurons (1 of 83) were reward-predictive in the late stage, but not in the early stage. In the striosome group, only 2% (2 of 122) of the neurons were reward-predictive in both learning stages. Therefore, total proportion of the striosome group was not significantly different from that of the control group, while it was larger in the late stage (early: 10%, striosome, and 13%, control, *p* = 0.45; late: 11%, striosome, and 1%, control, *p* = 0.0056, χ^2^ test; Fig. 4*B*). Compared with the early stage, reward-predictive neurons in the control group decreased in the late stage (*p* = 0.0027). Moreover, the majority of reward-predictive neurons had positive regression coefficients to *Vr* (early: 50%, striosome, and 82%, control; late: 93%, striosome, and 100%, control).

Furthermore, to study neural representation of expected reward at the population level, we performed a decoding analysis of forthcoming reward size from simultaneously recorded neuronal activities. Since the numbers of simultaneously recorded neurons were different in each mouse, we randomly selected *n* neurons from each simultaneously recorded population and used their activities during the CS-delay period for linear regression of forthcoming reward size (Fig. 4*C*; see Materials and Methods). We varied the subpopulation size *n* from 1 to 10 and for each *n*, we took 100 random combinations of neurons and compared the mean squared errors (MSEs) for striosome and control groups in early and late stages (Fig. 4*D*). The results indicated that MSEs of the striosome group were significantly larger in the early stage and smaller in the late stage than those of the control group (early: *p* = 1.1e-04, late: *p* = 0.0020, paired *t* test).

These analyses of reward-predictive neural activities revealed that neurons in striosomes represent reward values of odor stimuli in specific learning stages, and that reward-predictive striosomal neurons are more dominant in the late learning stage.

### Air-puff-predictive neural activities

**Figure 5.**
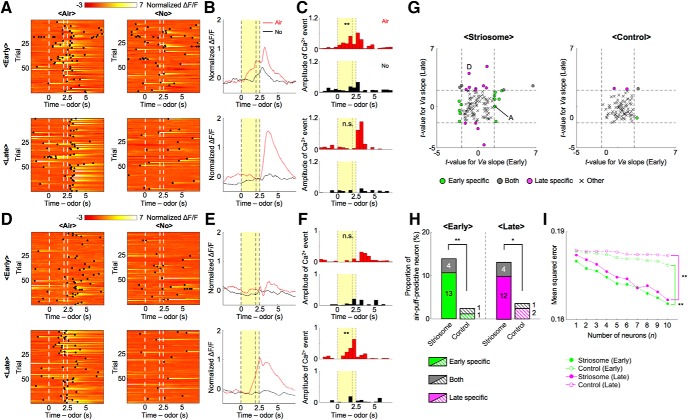
During each learning stage, different neural ensembles in the striosome predicted air-puff stimuli. ***A***, Normalized ΔF/F of a striosomal neuron showing air-puff-predictive activities specifically in the early stage. Black dots indicate detected Ca^2+^ events. ***B***, ***C***, Averaged ΔF/F and Ca^2+^ events of the striosomal neuron illustrated in ***A***. Yellow areas show the CS-delay period; **p < 0.01, n.s.: *p* ≥ 0.05, two-sample *t* test. ***D***, Normalized ΔF/F of another striosomal neuron showing air-puff-predictive activities specifically in the late stage. ***E***, ***F***, Averaged ΔF/F and Ca^2+^ events of the striosomal neuron illustrated in ***D***; **p < 0.01, n.s.: *p* ≥ 0.05. ***G***, Scatter plots of t-values for regression coefficients of prediction of air puff (*Va*) in each learning stage. Dashed lines indicate levels of significant *Va* slope at *p* = 0.05. Letters A and D indicate the example neurons in ***A***, ***D***. ***H***, Proportions of air-puff-predictive neurons in each learning stage. Numbers in bars indicate actual counts of air-puff-predictive neurons. **p < 0.01, *p < 0.05, χ^2^ test. ***I***, MSEs between actual and decoded air-puff values at each number of neurons used for analyses; **p < 0.01, paired *t* test.

We next examined whether recorded neurons responded to air-puff-predictive odor stimuli. In the early stage, the normalized ΔF/F of a representative striosomal neuron (Fig. 5*A*,*B*) rose with the presentation of odor stimuli associated with an air puff, whereas this rise was not observed in the no-reward condition. The sum of amplitudes of Ca^2+^ events during the CS-delay period in the early stage was significantly larger in the air-puff condition than in the no-reward condition (*p* = 0.036, two-sample *t* test; Fig. 5*C*). On the other hand, CS-delay period activity in the late stage showed no significant difference between the air-puff condition and the no-reward condition (*p* = 0.98) as the ΔF/F response to odor stimuli associated with the air puff became weak.

Contrastingly, the sum of amplitudes of Ca^2+^ events in another striosomal neuron (Fig. 5*D*,*E*) during the CS-delay period in the early stage showed no significant difference between air-puff and no-reward conditions (*p* = 0.35), while amplitudes in the late stage were significantly larger in the air-puff condition than in the no-reward condition (*p* = 1.0e-04; Fig. 5*F*). Neurons in which the sum of amplitudes of Ca^2+^ events during the CS-delay period differed significantly between the air-puff and no-reward conditions in one of the learning stages were also found in the control group.

Next, we analyzed air-puff-predictive activity using the predicted delivery of an air puff (*Va*) as the regressor. As in the case of reward-predictive activities, air-puff-predictive activities were observed specifically in one learning stage or the other (Fig. 5*G*). 11% of striosomal neurons (13 of 122) and 1% of control neurons (1 of 83) were air-puff-predictive in the early stage, but not in the late stage. On the other hand, 10% of striosomal neurons (12 of 122) and 2% of control neurons (2 of 83) were air-puff-predictive in the late stage, but not in the early stage. 3% of striosomal neurons (4 of 122) and 1% of control neurons (1 of 83) were air-puff-predictive in both learning stages. This means that total proportions of the striosome group were significantly larger than those of the control group in both learning stages (early: 14%, striosome, and 2%, control, *p* = 0.0052; late: 13%, striosome, and 4%, control, *p* = 0.021, χ^2^ test; Fig. 5*H*). Moreover, the majority of air-puff-predictive striosomal neurons had positive regression coefficients to *Air* (early: 58%, striosome, and 100%, control; late: 75%, striosome, and 100%, control).

Furthermore, to compare the population neural coding of expected aversive stimulus between two groups, we decoded forthcoming air-puff stimuli from the activities of various sizes of subpopulations of simultaneously recorded neurons (Fig. 5*I*). In both learning stages, MSEs of the striosome group were significantly smaller than those of the control group (early: *p* = 1.1e-05, late: *p* = 2.1e-04, paired *t* test). These analyses of air-puff-predictive neural activities showed that neurons in striosomes also represent aversive values of odor stimuli in learning-stage-specific ways, as is the case with reward values, and suggest that aversive values are more strongly encoded in the striosomes than in the matrix.

### Reward- and air-puff-responsive neural activities

**Figure 6.**
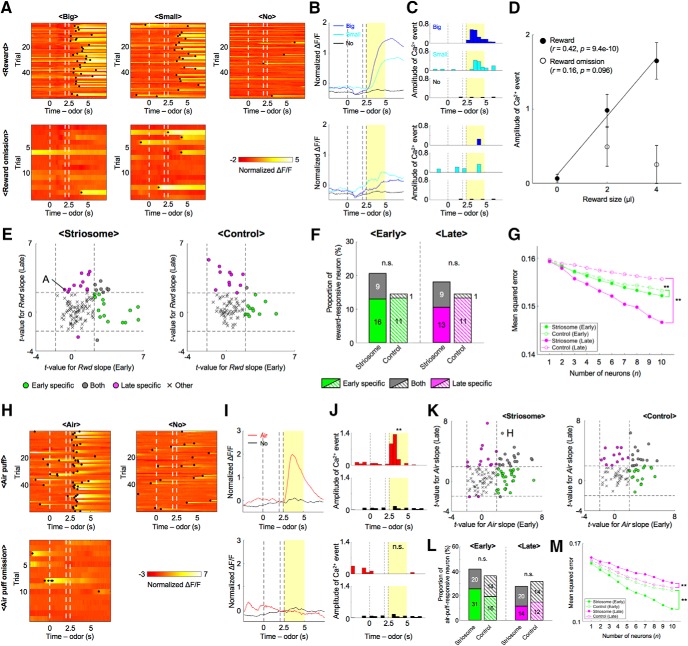
Both rewards and air puffs activated striosomal neurons. ***A***, Normalized ΔF/F of a striosomal neuron showing reward-responsive activities. This is ΔF/F in the late stage. Black dots indicate detected Ca^2+^ events. ***B***, ***C***, Averaged ΔF/F and Ca^2+^ events of the striosomal neuron illustrated in ***A***. Yellow areas show the US period. ***D***, Amplitudes of US period Ca^2+^ events of the striosomal neuron illustrated in ***A*** were averaged over trials and plotted against reward size. In rewarded trials, Ca^2+^ events show a positive correlation with reward size (*r* = 0.42, *p* = 9.4e-10). On the other hand, there was no significant correlation in reward-omitted trials (*r* = 0.16, *p* = 0.096). Error bars and lines indicate SEs and regression lines. ***E***, Scatter plots of t-values for regression coefficients of delivery of reward (*Rwd*) in each learning stage. Dashed lines indicate levels of significant *Rwd* slope at *p* = 0.05. Letter A indicates the example neuron in ***A***. ***F***, Proportions of reward-responsive neurons in each learning stage. Numbers in bars indicate actual counts of reward-responsive neurons; n.s.: *p* ≥ 0.05, χ^2^ test. ***G*,** MSEs between actually received and decoded reward size at each number of neurons used for analyses; **p < 0.01, paired *t* test. ***H*,** Normalized ΔF/F of a striosomal neuron showing air-puff-responsive activities. This is also ΔF/F in the late stage. Black dots indicate detected Ca^2+^ events. ***I***, ***J***, Averaged ΔF/F and Ca^2+^ events of the striosomal neuron illustrated in ***H***; **p < 0.01, n.s.: *p* ≥ 0.05, two-sample *t* test. ***K***, Scatter plots of *t* values for regression coefficients of delivery of air puff (*Air*) in each learning stage. Dashed lines indicate levels of significant *Air* slope at *p* = 0.05. Letter H indicate example neurons in ***H***. ***L***, Proportions of air-puff-responsive neurons in each learning stage. Numbers in bars indicate actual counts of air-puff-responsive neurons. n.s.: *p* ≥ 0.05. ***M***, MSEs between actually received and decoded air-puff stimuli at each number of neurons used for analyses; **p < 0.01, paired *t* test.

The normalized ΔF/F of a representative striosomal neuron (Fig. 6*A*,*B*) rose with reward presentation, whereas that rise was not observed in the absence of a reward. The sum of amplitudes of Ca^2+^ events during the US period in rewarded trials was significantly larger in the big-reward condition than with no-reward (*p* = 1.35e-10, two-sample *t* test; Fig. 6*C*). On the other hand, amplitudes in reward-omitted trials did not differ significantly between big-reward and no-reward conditions (*p* = 0.25). Amplitude positively correlated with reward size in rewarded trials (*r* = 0.42, *p* = 9.4e-10; Fig. 6*D*), but not in reward-omitted trials (*r* = 0.16, *p* = 0.096). This indicates that striosomal neurons responded to the rewards themselves. Reward-responsive activities were also observed in neurons of the control group.

After subtracting the licking component (see Materials and Methods), regression analyses of the sum of amplitudes of Ca^2+^ events during the US period revealed that most reward-responsive neurons, which had significant regression coefficients to the acquired reward size *Rwd*, had learning-stage-specific properties, similar to those of reward-predictive neurons (Fig. 6*E*). A total of 13% of striosomal neurons (16 of 122) and 13% of control neurons (11 of 83) were reward responsive in the early stage but not in the late stage. On the other hand, 11% of striosomal neurons (13 of 122) and 13% of control neurons (11 of 83) were reward responsive in the late stage, but not in the early stage; 7% of all neurons showed reward-responsive activities in both learning stages in the striosome group (9 of 122), but only 1% in the control group (1 of 83). Therefore, total proportions of the striosome group were not significantly different from those of the control group in either learning stage (early: 20%, striosome, and 14%, control, *p* = 0.27; late: 18%, striosome, and 14%, control, *p* = 0.50, χ^2^ test; Fig. 6*F*).

In addition, we decoded acquired reward size from various numbers of simultaneously recorded neuronal activities during the US period (Fig. 6*G*). In both learning stages, MSEs of the striosome group were significantly smaller than those of the control group (early: *p* = 0.0034; late: *p* = 5.9e-04, paired *t* test). This decoding result also shows that the reward acquisition is more robustly presented by the striosome neurons.

The normalized ΔF/F of another striosomal neuron (Fig. 6*H*,*I*) rose with the presentation of an air-puff stimulus, whereas this rise was not observed without the air puff. The sum of amplitudes of Ca^2+^ events during the US period was significantly larger in the air-puff condition than in the no-reward condition (*p* = 1.49e-08; Fig. 6*J*), whereas the response in the air-puff-omitted trials was not significantly different from that in the no-reward condition (*p* = 0.28). This indicated that the striosomal neuron respond to the air-puff stimulus itself. The air-puff-responsive activities were observed in neurons of the control group as well.

We analyzed air-puff-responsive activity using received air puff (*Air*) as a regressor in much the same way as with reward-responsive activities (Fig. 6*K*). 25% (31 of 122) of striosomal neurons and 19% of control neurons (16 of 83) were air-puff responsive in the early stage, but not in the late stage. On the other hand, 11% of striosomal neurons (14 of 122) and 14% of control neurons (12 of 83) were air-puff responsive in the late stage, but not in the early stage. 16% of striosomal neurons (20 of 122) and 17% of control neurons (14 of 83) were air-puff responsive in both learning stages. This means that the two groups did not differ significantly in total proportions of air-puff-responsive neurons in either learning stage (early: 42%, striosome, and 36%, control, *p* = 0.42; late: 28%, striosome, and 31%, control, *p* = 0.59, χ^2^ test; Fig. 6*L*). Finally, we decoded received air-puff stimuli from various numbers of simultaneously recorded neuronal activities during the US period (Fig. 6*M*). MSEs of the striosome group were significantly larger in the early stage and smaller in the late stage than those of the control group (early: *p* = 6.2e-04; late: 3.2e-06, paired *t* test).

These results indicate that some striosomal neurons respond directly to reward or air-puff stimuli.

## Discussion

We performed selective *in vivo* calcium imaging of neurons in striosomes and monitored neural activities of mice performing a classical odor-conditioning task. To the best of our knowledge, this is the first report to characterize striosomal neuronal activities of living animals (Note: During the final revisions of this paper, another paper on selective imaging of striosomal and matrix neurons was published (Bloem et al., 2017)). The major findings were as follows. (1) Striosomal neurons showed reward- or air-puff-predictive activities; therefore, they encoded the values of odor stimuli. (2) Most reward or air-puff-predictive activities were specific to early or late learning stages. (3) Some striosomal neurons responded to presentation of a reward or an air puff. (4) Striosomal neurons have more significant roles in reward and air-puff prediction than randomly recorded striatal neurons.

### Predictive neural activities in striosomes

Although previous electrophysiological studies reported that striatal neurons represent value information (Samejima et al., 2005; Ito and Doya, 2009, 2015), they did not distinguish between striosomal and matrix neurons. In this study, we found that neurons in striosomes show reward- or air-puff-predictive activities that matched the definition of value, both by regression of single neuron activities and by decoding from population activities. We also found ∼10% of non-selectively recorded neurons in the DMS showed reward-predictive activities in the early stage. This proportion is consistent with a recent electrophysiological study (Ito and Doya, 2015). Since the licking frequency in cue period correlated with forthcoming reward size, it was possible that reward-predictive striosomal activity might represent motor behavior instead of reward size expected from odor stimuli. However, those activities represented the reward size even after removing the effects of licking. Thus, the striosome encodes values of odor stimuli.

This result that striosomal neurons encode values of present sensory states, supports reinforcement learning models that postulate that striosomal neurons learn state values (Barto, 1995; Doya, 2000, 2002). These models postulated that matrix neurons are involved in either action selection (actor) or action value learning. An alternative view, based on human brain imaging or lesion experiments, is that the dorsal and ventral striatum assume the roles of actor and critic, respectively (O'Doherty et al., 2004). However, the striosomes comprise a larger portion of the ventral striatum than of the dorsal striatum; whereas the matrix constitutes a smaller portion of the ventral striatum and a larger portion of the dorsal striatum (Gerfen, 1992). Therefore, the striosome-matrix difference may contribute to ventral-dorsal functional differences. A recent rabies tracing study indicated that both striosomal and matrix neurons project to dopaminergic neurons, with a higher density of SNc projecting neurons in the striosome, but a larger number in the matrix, given its larger volume (Smith et al., 2016). This new finding raises the possibility that matrix neurons are also directly involved in computation of reward prediction error signals. To test those hypotheses, we will need to record and analyze the activities of striosomal and matrix neurons during an operant conditioning task that involves choices between multiple actions. It would also be desirable to record selectively not only striosomal neurons, but also matrix neurons from the ventral, dorsomedial and dorsolateral striatum.

In both learning stages, the proportion of air-puff-predictive neurons was larger in the striosomes than in the control. Air-puff stimuli are widely used as aversive stimuli in rodents and known to cause avoidance behaviors such as predictive eye blinks (Cohen et al., 2012; Heiney et al., 2014; Piochon et al., 2014; Kloth et al., 2015). A recent study revealed anatomic connections to striosomes from the bed nucleus of the stria terminalis (Smith et al., 2016), which is known to be involved in fear or anxiety (Jennings et al., 2013; Kim et al., 2013). Furthermore, optogenetic inhibition of axon terminals of prefrontal neurons projecting to the striosomes reduced sensitivity to aversive light exposure in a cost-benefit conflict situation (Friedman et al., 2015). Air-puff-predictive neurons in striosomes might link aversive signals to avoidance behaviors through their projection to the SNr and the internal globus pallidus and fear or anxiety through their projection to the stria terminalis.

In the Sepw1-Cre mouse line used in this study, 83.2% of Cre-expressing neurons were D1 medium spiny neurons (MSNs), projecting monosynaptically to dopaminergic neurons in the SNc, while matrix neurons that do not express Cre had no such projections (Smith et al., 2016). It was shown in the same Sepw1-Cre line that striatonigral fibers originating from the striosome form bouquet-like arborizations innervating clusters of dopamine-containing neurons with tightly bundled dendrites (Crittenden et al., 2016). Therefore, it is expected that the majority of striosomal neurons that showed reward- and air-puff-predictive activities in this study have monosynaptic projections to dopaminergic neurons in the SNc, which encode reward-prediction errors (Schultz et al., 1997). Our present discovery that the majority of reward-predictive striosomal neurons showed activities positively correlated with reward values suggests that they contribute to subtraction of predicted reward in computing reward prediction errors. On the other hand, striosomal neuronal activities that were correlated negatively with reward or positively with air puffs might contribute to computation of saliency, including both reward and aversive information, which is represented by a subset of dopaminergic neurons (Matsumoto and Hikosaka, 2009).

### Learning-stage-specific neural ensembles coding value information

Since the endoscopic *in vivo* calcium imaging method made it possible to observe activities of the same neurons over long periods, we were able to compare value representations of each striatal neuron across learning stages. It was an unexpected finding that reward- or air-puff-predictive activities observed in the early stage disappeared in the late stage. It was also surprising that there were few neurons that showed reward- or air-puff-predictive activities in both early and late learning stages. This result indicates that value-coding neurons form unique ensembles depending on the learning stage. Combined with the finding of Thorn et al. (2010) that population activities of the striatum change with learning, the ensemble representation of value information in the early stage might contribute to goal-directed behavior, while that in the late stage might support habitual behavior.

### Differences in reward-related neural coding in striosomes and matrix

Different parts of the striatum, especially near its ventromedial to dorsolateral axis, have different roles in goal-directed and habitual behaviors (Pennartz et al., 2009). It was reported that population activities of DMS neurons become weaker after acquisition of habitual behavior (Thorn et al., 2010). In this study, we implanted endoscopes in the DMS and monitored their neural activities during reward-based learning. Our regression analyses show that the number of reward-predictive neurons in the control group in the late stage decreased from that in the early stage. This is consistent with the result of non-selective recording of DMS neurons. In the late stage, the proportion of reward-predictive neurons was larger in the striosome group than in the control group. Our decoding analyses also showed that population neural activities of striosomes represented expected rewards more strongly than the control in the late stage. It is expected that recorded neural activities from the control group are mostly derived from the matrix, since roughly 85% of striatum neurons belong to the matrix. This suggests a possibility that striosomal neurons assume a more dominant role in reward prediction after habituation than do matrix neurons. On the other hand, roughly 80% of neurons in striosomes are D1-MSNs and another 20% are D2-MSNs, whereas the proportion is around 50%-50% in matrix (Fujiyama et al., 2011). Therefore, the differences between the striosome and control groups may reflect the difference in D1/D2 percentages.

Our finding of reward- and air-puff-predictive activities of neurons in striosomes contributes to understanding of mechanisms of reinforcement learning in the brain. The next important issues to clarify are whether striosomal neurons encode the state value rather than the action value in a choice task, and to test whether and how striosomal neurons contribute to computation of reward-prediction error.

## Synthesis

Reviewing Editor: Thomas McHugh, RIKEN Brain Science Institute Decisions are customarily a result of the Reviewing Editor and the peer reviewers coming together and discussing their recommendations until a consensus is reached. When revisions are invited, a fact-based synthesis statement explaining their decision and outlining what is needed to prepare a revision will be listed below. The following reviewer(s) agreed to reveal their identity: Min Jung, Xin Jin.

Summary and Synthesis

In this study, the authors employ a mouse line selectively expressing Cre in striosomal neurons and endoscopic calcium imaging to examine striosomal neuronal activities during a Pavlovian learning task under head-fixed conditions. The major findings are that striosomal neurons encode state value that is specific to early or late learning stage, with more striosomal neurons encoding value in the late stage of learning compared to putative non-striosomal neurons. They also showed that striosomal neurons are responsive to appetitive and aversive stimuli. These finding are novel and interesting and the conclusions have a potential to stimulate further research in the field.

Both referees found this to be a well conducted and novel study that will be of use to the field. Following our discussion they have agreed on several points regarding both the analyses and discussion that the authors must address prior to publication.

There are six main points the authors should address in their revised manuscript.

1. The authors need to include lick frequency as a factor in their regression analyses both during delay and reward periods to control for motor-driven responses.

Reviewer #1 states: As the authors mentioned in the manuscript, striatal neuronal activity is closely related to motor activity. Since licks prior to reward may have affected neuronal responses, lick frequency must be included in the regression model in addition to reward. The authors showed the results of the regression analysis that included lick frequency as an additional analysis. However, all the main figures and data must be presented with the regression analysis that included lick frequency as an independent variable. In addition, lick frequency must be included in regression formula in the reward period. Because there is a possibility that lick frequency may differ with the amount of reward. Besides, lick rates after big reward seems to be higher than small reward in example shown in Figure 1C.

2. The authors need to modify their regression analysis of reward to dissociated outcome and value driven responses

Reviewer #1 states: Previous studies have shown that striatal neurons carry both value and outcome information during outcome period. I'm not sure how the authors dissociated outcome- and value-dependent neural activity during the reward period. I think both parameters should be included in the regression model to analyze neural activity in the reward period. It is unclear how exactly they coded the outcomes in the regression. Were reward omission trials included in this analysis?

3. The authors should provide examples of histology demonstrating GRIN lens placement and viral expression.

Reviewer #1 states: The authors claimed that they have inserted GRIN lens in the dorsomedial striatum. However, there are no figures showing the proper placement of the lens besides the cartoons shown in Figure 2. Lens location and the extent of virus expression must be shown.

4. The authors should discuss the findings in relation to various reinforcement learning models.

Reviewer #2 states: One major theory in the field of reinforcement learning model of basal ganglia is that the patch neurons encode the state values and matrix performs the action selection. Given the current results that a rather low proportion (~10%) of patch neurons actually encoding cue values, it will be hugely beneficial to the audiences if the authors could discuss their results in relation to the various models in the reinforcement learning field, and the potential revision on some of the dominant views.

5. In light of the recent rabies tracing data the authors should discuss the possibility that one subpopulation of SPNs in both the patch and matrix represent values, and the action selection is implemented by other subpopulations of patch and/or matrix neurons or downstream.

Reviewer #2 states: Recent rabies tracing results from multiple labs seemly indicated that both patch and matrix neurons project to dopaminergic neurons (Watabe-Uchida et al. 2012; Smith et al. 2016). As the authors stated, around 80% patch neurons are D1 and others are D2-SPNs, and the ratio is 50-50% in matrix. It means while the proportion of matrix neurons encoding cue values are low, there might be still significant amount of information sent from matrix D1-SPNs to dopaminergic neurons. Is it possible that one subpopulation of SPNs in both the patch and matrix represent values, and the action selection is implemented by other subpopulations of patch and/or matrix neurons or downstream?

6. The authors should discuss how dopaminergic neurons might incorporate the appetitive and aversive signals for learning, given striatal patch neurons encoding them both in the positive direction

Reviewer #2 states: The authors should discuss how dopaminergic neurons might incorporate the appetitive and aversive signals for learning, given striatal patch neurons encoding them both in the positive direction (Fig. 6).

## Author Response

Here are our responses to the six comments:

#1. The authors need to include lick frequency as a factor in their regression analyses both during delay and reward periods to control for motor-driven responses.

As Reviewer #1 requested, we performed regression analyses, including lick frequency, as described in the new “Experimental Design and Statistical Analysis” section of the Methods. Figures 4A, B, 5G, H, 6E, F, K, and L are revised accordingly. With the inclusion of lick frequency, the numbers of reward-predictive neurons in the control group in the early state decreased from 18 to 10, so that the proportions of reward predictive neurons in the striosome and control groups in the early learning stage are not significantly different. However, in the late learning stage, the proportion of reward-predictive neurons in the striosomes was significantly larger than in the control, consistent with the original results. The proportion of reward-responsive neurons in the striosome group also decreased in the late stage, so that the proportions were not significantly different between the two groups in either learning stage.

#2. The authors need to modify their regression analysis of reward to dissociated outcome and value driven responses.

In our previous manuscript, Rwd(i) and Air(i) represented reward prediction (value) in the CS-delay period and reward delivered (outcome) in the US period, while their values differ in reward/air puff omission trials. To avoid confusion, we revised the names of variables in regression models in the “Materials and Methods - Experimental Design and Statistical Analysis” section. We now use different variables, Vr and Va for the values and Rwd and Air for outcomes. We did not include Vr and Va for regression during the US period, because according to our paradigm, Vr and Rwd, and Va and Air, were highly correlated, with only 20% omission trials.

#3. The authors should provide examples of histology demonstrating GRIN lens placement and viral expression.

We included an example of histology demonstrating GRIN lens placement and viral expression in Figure 2G.

#4. The authors should discuss the findings in relation to various reinforcement learning models.

We discussed our results in light of three major theoretical models of reinforcement learning in the basal ganglia, namely, the actor-critic model, the state-action value model, and the dorsal-ventral striatum model in the “Discussion - Predictive neural activities in striosomes” section.

#5. In light of the recent rabies tracing data the authors should discuss the possibility that one subpopulation of SPNs in both the patch and matrix represent values, and the action selection is implemented by other subpopulations of patch and/or matrix neurons or downstream.

Since our recording did not discriminate D1- and D2-SPN subpopulations in the striosomes in this study, we only briefly discussed our present results with the recent rabies tracing data in the “Discussion - Predictive neural activities in striosomes” section.

#6. The authors should discuss how dopaminergic neurons might incorporate the appetitive and aversive signals for learning, given striatal patch neurons encoding them both in the positive direction.

We have discussed a possible role of striosomal signals for learning in association with reward prediction error and saliency signals of dopaminergic neurons in the “Discussion - Predictive neural activities in striosomes” section.
